# Calculation of the Gibbs–Donnan factors for multi-ion solutions with non-permeating charge on both sides of a permselective membrane

**DOI:** 10.1038/s41598-021-00899-y

**Published:** 2021-11-12

**Authors:** Jacek Waniewski, Mauro Pietribiasi, Leszek Pstras

**Affiliations:** Nalecz Institute of Biocybernetics and Biomedical Engineering, Polish Academy of Sciences, Trojdena 4, 02-109 Warsaw, Poland

**Keywords:** Biomedical engineering, Membranes, Circulation

## Abstract

Separation of two ionic solutions with a permselective membrane that is impermeable to some of the ions leads to an uneven distribution of permeating ions on the two sides of the membrane described by the Gibbs–Donnan (G–D) equilibrium with the G–D factors relating ion concentrations in the two solutions. Here, we present a method of calculating the G–D factors for ideal electroneutral multi-ion solutions with different total charge of non-permeating species on each side of a permselective membrane separating two compartments. We discuss some special cases of G–D equilibrium for which an analytical solution may be found, and we prove the transitivity of G–D factors for multi-ion solutions in several compartments interconnected by permselective membranes. We show a few examples of calculation of the G–D factors for both simple and complex solutions, including the case of human blood plasma and interstitial fluid separated by capillary walls. The article is accompanied by an online tool that enables the calculation of the G–D factors and the equilibrium concentrations for multi-ion solutions with various composition in terms of permeating ions and non-permeating charge, according to the presented method.

## Introduction

The Gibbs–Donnan theory describes the equilibrium conditions for ion solutions separated by a permselective (semipermeable) membrane when one of the solutions contains species that cannot pass through the membrane (non-permeating charge), which distorts the distribution of permeating ions on the two sides of the membrane^1–4^. The restriction of ion permeability may be due to its size, as for macromolecules, such as proteins, or due to the membrane charge, as in ion exchange membranes^5,6^. The theory proposed by J.W. Gibbs in 1878 and proved experimentally by F.G. Donnan in 1911^2^ has practical applications in several fields, e.g. material and membrane sciences^7–9^, chromatography^10^, and biology^11–13^. In medicine and biomedical engineering the Gibbs–Donnan theory is most often used to describe the composition of blood plasma (rich in proteins) separated by a capillary wall from the interstitial fluid (with low protein content)^14–17^ or by a dialyzer or filter membrane from a protein-free dialysis fluid^18–20^. The theory introduces a parameter, called the Gibbs–Donnan (G–D) factor (or coefficient), that, when multiplied by the known concentration of the given ion in the macromolecule-rich fluid (such as plasma), provides its equilibrium concentration in the macromolecule-free fluid (such as dialysis fluid). The G–D factor depends on the charge and concentrations of both permeating and non-permeating species^1^. The proper estimation of this factor is important for the description of transport processes across the membrane, both in equilibrium and under non-steady conditions^1,21^.

**Figure 1:**
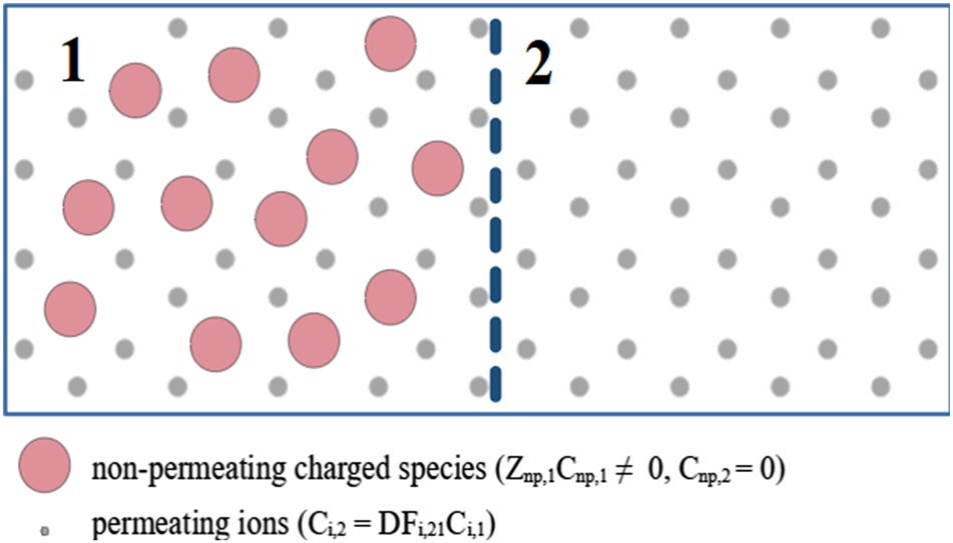
Gibbs–Donnan equilibrium: compartment 1 and compartment 2 are separated by a permselective membrane; non-permeating charged species (np) are present only in compartment 1. Symbols: *Z* charge number, *C* concentration, *DF* Gibbs–Donnan factor.

**Figure 2:**
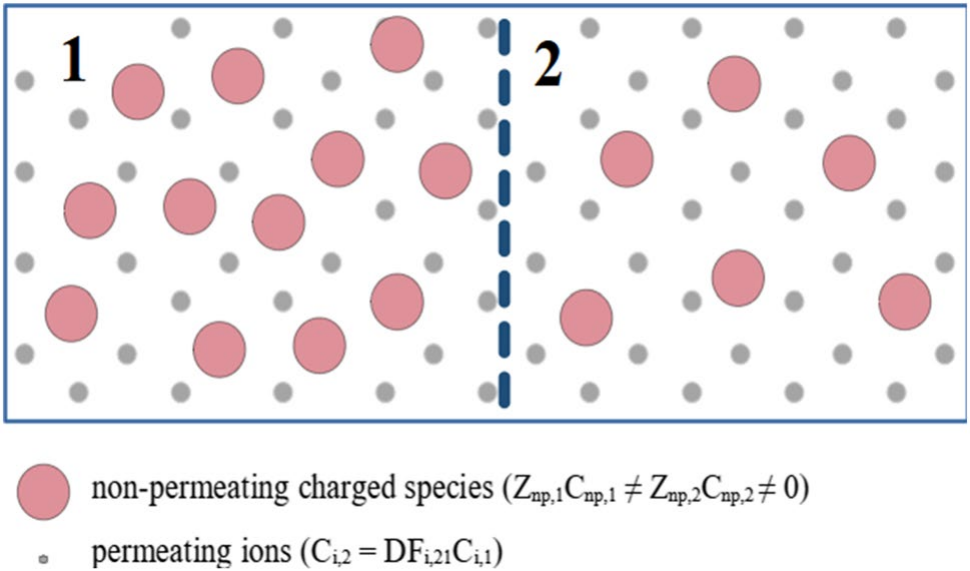
Extended Gibbs–Donnan equilibrium: compartment 1 and compartment 2 are separated by a permselective membrane; non-permeating charged species (np) are present in both compartments. Symbols: *Z* charge number, *C* concentration, *DF* Gibbs–Donnan factor.

The G–D theory is typically applied to simple sets of ions, such as dissociated NaCl or CaCl_2_, with the charged macromolecules present only on one side of a permselective membrane^1^, see Fig. 1. In real life, however, more complex multi-ion cases are present, often with the macromolecules on both sides of the membrane at different concentration (and therefore different total charge), see Fig. 2. For instance, the protein-rich blood plasma contains several small ions with different charge numbers^17^ and is separated by the capillary walls from the interstitial fluid, which also contains some charged proteins. This study presents and discusses the method for calculation of the G–D factors for such more complex cases and is accompanied by an MS Excel-based calculator of the G–D equilibrium for any mixture of ions (see Supplementary material).

## Theoretical derivations

### The Gibbs–Donnan theory for multi-ion solutions

Let us consider two compartments separated by a selectively permeable membrane. Each compartment contains an aqueous solution with multiple charged species, some of which the membrane is permeable to (permeating ions) and some other to which the membrane is not permeable (non-permeating charged species).

The solution in each compartment contains n permeating ions with charge number z_i_ and molar concentration c_i_, i = 1,2,…n. In total, all permeating ions have s different values of the charge number v_α_, α = 1,2,…s. The non-permeating (np) species in each solution are described by Z_np_ C_np_, where Z_np_ is their average (concentration-weighted) charge number and C_np_ is their total molar concentration (fixed in each compartment). Note that this is an aggregated description of non-permeating species, as in general there might be multiple non-permeating species with different charge numbers and concentrations; therefore, Z_np_ C_np_ denotes the sum of the respective ZC products over all non-permeating species.

The electroneutrality of the solution in each compartment requires:

1 $$\sum\limits_{{\text{i = 1}}}^{{\text{n}}} {{\text{z}}_{{\text{i}}} } {\text{c}}_{{\text{i,1}}} {\text{ + Z}}_{{\text{np,1}}} {\text{C}}_{{\text{np,1}}} { = 0}$$

for the compartment 1, and

2 $$\sum\limits_{{{\text{i = 1}}}}^{{\text{n}}} {{\text{z}}_{{\text{i}}} } {\text{c}}_{{{\text{i,2}}}} {\text{ + Z}}_{{{\text{np,2}}}} {\text{C}}_{{{\text{np,2}}}} {\text{ = 0}}$$

for the compartment 2.

The product of ion charge number and concentration, zc, may be referred to as ionic equivalent (for example in mEq/L). Thus, ionic equivalent multiplied by a constant that describes the charge of the corresponding amount of elementary positive charges (e.g. the charge of a millimole of monovalent cations), gives us the charge density of the ion (including the sign of charge).

The equilibrium of two multi-ion solutions separated by a permselective membrane requires the intercompartment equilibration of the electrochemical potentials of each permeating ion as well as the equilibration of the chemical potential of the solvent in both compartments. Let us assume that the considered permselective membrane is either uncharged (size-selective) or that it has a much higher charge density compared to the bulk charge of either of the solutions (charge-selective). Let us also assume that there is no electrochemical gradient across the membrane caused by mechanisms other than the G–D effect discussed here (e.g. active transport of ions across biological cell membranes). In particular, we assume that there is no chemical gradient caused by a hydrostatic or hydraulic pressure difference across the membrane (here, we consider only the electro-diffusive transport of ions and not the possible osmotic shifts of the solvent across the membrane).

At the state of equilibrium, the activities of a permeating ion i (a_i_) in two compartments separated by a permselective membrane are related to the Nernst potential as follows:

3 $${\text{V}}^{{{\text{Nernst}}}} { = } - \frac{{{\text{RT}}}}{{{\text{z}}_{{\text{i}}} {\text{F}}}}{\text{ln}}\left( {\frac{{{\text{a}}_{{\text{i,2}}} }}{{{\text{a}}_{{\text{i,1}}} }}} \right)$$

where R is the perfect gas constant, F the Faraday constant, and T the absolute temperature of the mixture.

The ratios of the activities of each permeating ion in compartments 1 and 2 are hence related as follows:

4 $$\left( {\frac{{{\text{a}}_{{\text{i,2}}} }}{{{\text{a}}_{{\text{i,1}}} }}} \right)^{{{\text{1/z}}_{{\text{i}}} }} {\text{ = exp}}\left( { - \frac{{{\text{FV}}^{{{\text{Nernst}}}} }}{{{\text{RT}}}}} \right){\text{ = const}}$$

The above equations hold for individual ion species, but one can also sum up the activities of all ions with the same charge number v_α_, ${\text{A}}_{{\upalpha }} { = }\sum\limits_{{{\text{z}}_{{\text{i}}} {\text{ = v}}_{{\upalpha }} }} {{\text{a}}_{{\text{i}}} }$, and then:

5 $$\left( {\frac{{{\text{A}}_{{{{\upalpha ,2}}}} }}{{{\text{A}}_{{{{\upalpha ,1}}}} }}} \right)^{{{\text{1/v}}_{{\upalpha }} }} {\text{ = exp}}\left( {{ - }\frac{{{\text{FV}}^{{{\text{Nernst}}}} }}{{{\text{RT}}}}} \right){\text{ = const}}$$

for all charge numbers v_α_. This may be shown formally as follows:

6 $$\begin{gathered} \left( {\frac{{{\text{A}}_{{{{\upalpha ,2}}}} }}{{{\text{A}}_{{{{\upalpha ,1}}}} }}} \right)^{{{\text{1/v}}_{{\upalpha }} }} { = }\left( {\frac{{\sum\limits_{{{\text{z}}_{{\text{i}}} {\text{ = v}}_{{\upalpha }} }} {{\text{a}}_{{\text{i,2}}} } }}{{\sum\limits_{{{\text{z}}_{{\text{i}}} {\text{ = v}}_{{\upalpha }} }} {{\text{a}}_{{\text{i,1}}} } }}} \right)^{{{\text{1/v}}_{{\upalpha }} }} { = }\left( {\frac{{\sum\limits_{{{\text{z}}_{{\text{i}}} {\text{ = v}}_{{\upalpha }} }} {\frac{{{\text{a}}_{{\text{i,2}}} }}{{{\text{a}}_{{\text{i,1}}} }}{\text{a}}_{{\text{i,1}}} } }}{{\sum\limits_{{{\text{z}}_{{\text{i}}} {\text{ = v}}_{{\upalpha }} }} {{\text{a}}_{{\text{i,1}}} } }}} \right)^{{{\text{1/v}}_{{\upalpha }} }} { = }\left( {\frac{{{\text{exp}}\left( {{ - }\frac{{{\text{v}}_{{\upalpha }} {\text{FV}}^{{{\text{Nernst}}}} }}{{{\text{RT}}}}} \right)\sum\limits_{{{\text{z}}_{{\text{i}}} {\text{ = v}}_{{\upalpha }} }} {{\text{a}}_{{\text{i,1}}} } }}{{\sum\limits_{{{\text{z}}_{{\text{i}}} {\text{ = v}}_{{\upalpha }} }} {{\text{a}}_{{\text{i,1}}} } }}} \right)^{{{\text{1/v}}_{{\upalpha }} }} { = } \hfill \\ \left( {{\text{exp}}\left( {{ - }\frac{{{\text{v}}_{{\upalpha }} {\text{FV}}^{{{\text{Nernst}}}} }}{{{\text{RT}}}}} \right)} \right)^{{{\text{1/v}}_{{\upalpha }} }} {\text{ = exp}}\left( {{ - }\frac{{{\text{FV}}^{{{\text{Nernst}}}} }}{{{\text{RT}}}}} \right) \hfill \\ \end{gathered}$$

From now on, we will assume ideal solutions in which ion activity is equal to ion concentration and independent from the concentrations of other ions in the mixture, a_i_ = c_i_ (for a general, non-ideal case, see Appendix A in the Supplementary material). We also assume that all considered permeating ions are present only in fully dissociated form, i.e., that they do not form any chemical compounds (pairs or complexes) with their counterions (for the description of a general case with the ions present in various chemical forms, see Appendix B in the Supplementary material). We also assume that none of the permeating ions are bound to non-permeating species; in other words, we assume that c_i_ denotes the concentration of the free-fraction of ion i in the mixture. Moreover, we assume that the permeating ions do not adsorb on the membrane. Finally, we assume that ions with the same charge number (e.g. Na^+^ and K^+^, or Mg^2+^ and Ca^2+^) interact with other charged species in the same way, which is a simplification of complex ion-ion interactions in non-ideal multi-ion solutions.

Based on the above assumptions and Eq. (5), for any two charge numbers v_α_ and v_β_ (α, β = 1,2,…s), we have:

7 $$\left( {\frac{{{\text{C}}_{{{{\upalpha ,2}}}} }}{{{\text{C}}_{{{{\upalpha ,1}}}} }}} \right)^{{{\text{1/v}}_{{\upalpha }} }} { = }\left( {\frac{{{\text{C}}_{{{{\beta ,2}}}} }}{{{\text{C}}_{{{{\beta ,1}}}} }}} \right)^{{{\text{1/v}}_{{\upbeta }} }}$$

where C_α_ is the total concentration of all ions with the same charge number v_α_, ${\text{C}}_{{\upalpha }} { = }\sum\limits_{{{\text{z}}_{{\text{i}}} {\text{ = v}}_{{\upalpha }} }} {{\text{c}}_{{\text{i}}} }$.

From Eqs. (1, 2) we have:

8 $$\sum\limits_{{{{\upalpha = 1}}}}^{{\text{s}}} {{\text{v}}_{{\upalpha }} } {\text{C}}_{{{{\upalpha ,1}}}} {\text{ + Z}}_{{\text{np,1}}} {\text{C}}_{{\text{np,1}}} { = 0}$$

9 $$\sum\limits_{{{{\upalpha = 1}}}}^{{\text{s}}} {{\text{v}}_{{\upalpha }} } {\text{C}}_{{{{\upalpha ,2}}}} {\text{ + Z}}_{{\text{np,2}}} {\text{C}}_{{\text{np,2}}} { = 0}$$

Let us assume now that we know the equilibrium concentrations of all permeating ions in compartment 1 and want to calculate the equilibrium concentrations in compartment 2. Let us also select all permeating ions with the charge number v_1_ as a reference species (note that this selection is arbitrary and that permeating ions with any charge number present in the mixture may be selected as a reference species). Then, using Eq. (7):

10 $${\text{C}}_{{{{\upalpha ,2}}}} { = }\left( {\frac{{{\text{C}}_{{1,2}} }}{{{\text{C}}_{{1,1}} }}} \right)^{{{\text{v}}_{{\upalpha }} {\text{/v}}_{{1}} }} {\text{C}}_{{{{\upalpha ,1}}}}$$

for all α = 1,2,…s.

Thus, we can reduce the problem to calculation of the ratio ${\text{x = C}}_{{1,2}} {\text{/C}}_{{1,1}}$ or, equivalently, calculation of C_1,2_. Using Eqs. (9, 10) we have:

11 $$\sum\limits_{{{{\upalpha = 1}}}}^{{\text{s}}} {{\text{x}}^{{{\text{v}}_{{\upalpha }} {\text{/v}}_{{1}} }} } {\upgamma }_{{\upalpha }} {{ + \upgamma }}_{{\text{np,2}}} { = 0}$$

where ${\upgamma }_{{\upalpha }} {\text{ = v}}_{{\upalpha }} {\text{C}}_{{{{\upalpha ,1}}}} {/}\left( {{\text{v}}_{{1}} {\text{C}}_{{1,1}} } \right)$ and ${\upgamma }_{{\text{np,2}}} {\text{ = Z}}_{{\text{np,2}}} {\text{C}}_{{\text{np,2}}} {/}\left( {{\text{v}}_{{1}} {\text{C}}_{{1,1}} } \right)$ are relative ionic equivalents versus the equivalent of the selected reference ion in compartment 1. Note that ${\upgamma }_{{1}} { = 1}$.

To avoid negative exponents in the above equation, let v_max_ be the maximal value of v_α_ and − v_α_. Then:

12 $$\sum\limits_{{{{\upalpha = 1}}}}^{{\text{s}}} {{\text{x}}^{{\left( {{\text{v}}_{{{\text{max}}}} {\text{ + v}}_{{\upalpha }} } \right){\text{/v}}_{{1}} }} } {\upgamma }_{{\upalpha }} {{ + \upgamma }}_{{\text{np,2}}} {\text{x}}^{{{\text{v}}_{{{\text{max}}}} {\text{/v}}_{{1}} }} { = 0}$$

or, for $${\text{y = x}}^{{{\text{1/v}}_{{1}} }}$$:

13 $$\sum\limits_{{{{\upalpha = 1}}}}^{{\text{s}}} {{\text{y}}^{{\left( {{\text{v}}_{{{\text{max}}}} {\text{ + v}}_{{\upalpha }} } \right)}} } {\upgamma }_{{\upalpha }} {{ + \upgamma }}_{{\text{np,2}}} {\text{y}}^{{{\text{v}}_{{{\text{max}}}} }} { = 0}$$

is the polynomial that can be solved in closed formulas for some simple mixtures of ions. For s = 1 one has ${{{\rm x} = - \upgamma }}_{{\text{np,2}}}$.

Furthermore, if s (the number of distinct charge numbers of permeating ions) is higher than 1, from Eq. (8) we get:

14 $${\upgamma }_{{\text{s}}} { = - }\left( {\sum\limits_{{{{\upalpha = 1}}}}^{{\text{s - 1}}} {{\upgamma }_{{\upalpha }} } {{ + \upgamma }}_{{\text{np,1}}} } \right)$$

where ${\upgamma }_{{\text{s}}} {\text{ = v}}_{{\text{s}}} {\text{C}}_{{\text{s,1}}} {/}\left( {{\text{v}}_{{1}} {\text{C}}_{{1,1}} } \right)$_,_ ${\upgamma }_{{\upalpha }} {\text{ = v}}_{{\upalpha }} {\text{C}}_{{{{\upalpha ,1}}}} {/}\left( {{\text{v}}_{{1}} {\text{C}}_{{1,1}} } \right)$, and ${\upgamma }_{{\text{np,1}}} {\text{ = Z}}_{{\text{np,1}}} {\text{C}}_{{\text{np,1}}} {/}\left( {{\text{v}}_{{1}} {\text{C}}_{{1,1}} } \right)$, and then:

15 $$\sum\limits_{{{{\upalpha = 1}}}}^{{\text{s - 1}}} {{\upgamma }_{{\upalpha }} {\text{x}}^{{\left( {{\text{v}}_{{{\text{max}}}} {\text{ + v}}_{{\upalpha }} } \right){\text{/v}}_{{1}} }} } { - }\left( {\sum\limits_{{{{\upalpha = 1}}}}^{{\text{s - 1}}} {{\upgamma }_{{\upalpha }} } {{ + \upgamma }}_{{\text{np,1}}} } \right){\text{x}}^{{\left( {{\text{v}}_{{{\text{max}}}} {\text{ + v}}_{{\text{s}}} } \right){\text{/v}}_{{1}} }} {{ + \upgamma }}_{{\text{np,2}}} {\text{x}}^{{{\text{v}}_{{{\text{max}}}} {\text{/v}}_{{1}} }} { = 0}$$

or

16 $$\sum\limits_{{{{\upalpha = 1}}}}^{{\text{s - 1}}} {{\upgamma }_{{\upalpha }} {\text{y}}^{{{\text{v}}_{{{\text{max}}}} {\text{ + v}}_{{\upalpha }} }} } { - }\left( {\sum\limits_{{{{\upalpha = 1}}}}^{{\text{s - 1}}} {{\upgamma }_{{\upalpha }} } {{ + \upgamma }}_{{\text{np,1}}} } \right){\text{y}}^{{{\text{v}}_{{{\text{max}}}} {\text{ + v}}_{{\text{s}}} }} {{ + \upgamma }}_{{\text{np,2}}} {\text{y}}^{{{\text{v}}_{{{\text{max}}}} }} { = 0}$$

The above equations are presented for the total concentrations ${\text{C}}_{{\upalpha }}$ of all permeating ions with the same charge number ${\text{v}}_{{\upalpha }}$ but they may also be rewritten for individual species concentrations ${\text{c}}_{{\text{i}}}$.

The Gibbs–Donnan factor (often referred to as Donnan factor) for ions with the charge number v_α_ is:

17 $${\text{DF}}_{{{{\upalpha ,21}}}} {\text{ = x}}^{{{\text{v}}_{{\upalpha }} {\text{/v}}_{{1}} }}$$

or:

18 $${\text{DF}}_{{{{\upalpha ,21}}}} {\text{ = y}}^{{{\text{v}}_{{\upalpha }} }}$$

and allows for calculation of ${\text{C}}_{{{{\upalpha ,2}}}}$ if ${\text{C}}_{{{{\upalpha ,1}}}}$ is known:

19 $${\text{C}}_{{{{\upalpha ,2}}}} {\text{ = DF}}_{{{{\upalpha ,21}}}} {\text{C}}_{{{{\upalpha ,1}}}}$$

The G–D factor depends on the overall composition of the ionic mixture, i.e. the number of different charge numbers of permeating ions, s, and their values z, and the relative ionic equivalents, γ. Therefore, for the given ionic mixture, the G–D factor has a scaling property and does not change if the ion concentrations change without modification of relative ionic equivalents. In general, the G–D factor depends on the relative ionic equivalents of all permeating and non-permeating species.

In the case of s = 1, i.e. with all permeating ions with the same charge number, from Eqs. (1, 2) we get:

20 $${\text{v}}_{{1}} {\text{C}}_{{1,1}} {\text{ + Z}}_{{\text{np,1}}} {\text{C}}_{{\text{np,1}}} { = 0}$$

21 $${\text{v}}_{{1}} {\text{C}}_{{1,2}} {\text{ + Z}}_{{\text{np,2}}} {\text{C}}_{{\text{np,2}}} { = 0}$$

and:

22 $${\text{DF}}_{{1,21}} { = }\frac{{{\text{C}}_{{1,2}} }}{{{\text{C}}_{{1,1}} }}{ = }\frac{{{\text{Z}}_{{\text{np,2}}} {\text{C}}_{{\text{np,2}}} }}{{{\text{Z}}_{{\text{np,1}}} {\text{C}}_{{\text{np,1}}} }}{ = }\frac{{{\upgamma }_{{\text{np,2}}} }}{{{\upgamma }_{{\text{np,1}}} }}{{ = - \upgamma }}_{{\text{np,2}}}$$

because, by Eq. (8), ${\upgamma }_{{\text{np,1}}} { = - 1}$ if ${\text{s = 1}}$ (compare Eqs. (12, 13), and the comment that follows them). Note that in this case ${\text{Z}}_{{\text{np,1}}} {\text{C}}_{{\text{np,1}}}$ and ${\text{Z}}_{{\text{np,2}}} {\text{C}}_{{\text{np,2}}}$ have the same sign, as it results from the above electroneutrality conditions for the two compartments.

## Transitivity of Gibbs–Donnan factors

**Figure 3:**
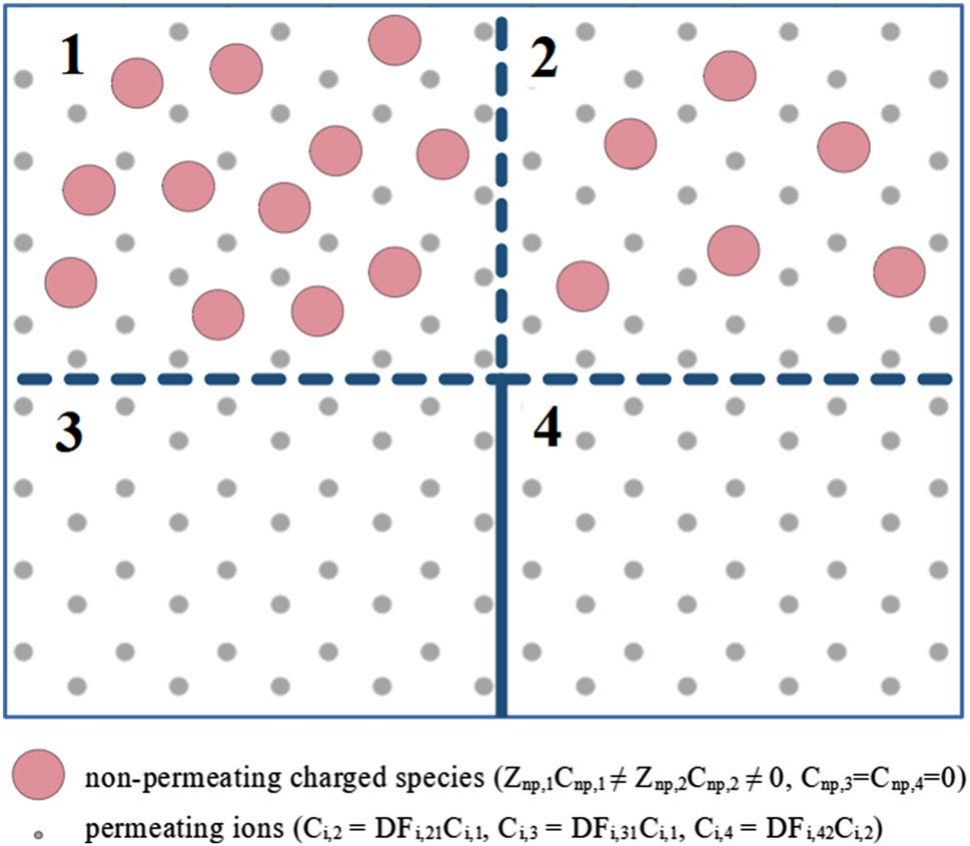
Transitivity of Gibbs–Donnan factors: compartments 1 and 2, 2 and 4, and 1 and 3 are separated by identical permselective membranes; non-permeating charged species (np) are present in compartments 1 and 2 only. In equilibrium, the concentrations of permeating ions in compartments 3 and 4 are equal, C_i,4_ = C_i,3_. The Gibbs–Donnan factors for equilibrium between compartments with non-permeating species, compartment 1 vs. 2, can be calculated using Gibbs–Donnan factors for compartments without non-permeating species, 1 vs. 3 and 2 vs. 4: DF_i,21_ = DF_i,31_ /DF_i,42_. Symbols: *Z* charge number, *C* concentration, *DF* Gibbs–Donnan factor.

Let us consider four compartments 1–4 arranged in series with permselective membranes (not permeable to certain charged species) separating compartment 1 from compartment 2, compartment 2 from compartment 4, and compartment 1 from compartment 3 (see Fig. 3).

The compartments 1 and 2 contain non-permeating charged species with the equivalents Z_np,1_C_np,1_ and Z_np,2_C_np,2_, respectively, whereas the compartments 3 and 4 do not contain non-permeating species. The whole system of compartments contains also a mixture of positively and negatively charged permeating ions. The G–D factor for ions with the charge number v_α_ and compartments k and r is defined as:

23 $${\text{C}}_{{{{\upalpha ,k}}}} {\text{ = x}}_{{{\text{kr}}}}^{{{\text{v}}_{{\upalpha }} {\text{/v}}_{{1}} }} {\text{C}}_{{{{\upalpha ,r}}}} {\text{ = DF}}_{{{{\upalpha ,kr}}}} {\text{C}}_{{{{\upalpha ,r}}}}$$

where $${\text{x}}_{{{\text{kr}}}} {\text{ = C}}_{{\text{1,k}}} {\text{/C}}_{{\text{1,r}}}$$.

Then, for each ${\upalpha }$: ${\text{C}}_{{{{\upalpha ,3}}}} {\text{ = C}}_{{{{\upalpha ,4}}}}$ and:

24 $${\text{DF}}_{{{{\upalpha ,21}}}} {\text{ = DF}}_{{{{\upalpha ,31}}}} {\text{DF}}_{{{{\upalpha ,24}}}}$$

or:

25 $${\text{DF}}_{{{{\upalpha ,21}}}} {\text{ = DF}}_{{{{\upalpha ,31}}}} {\text{/DF}}_{{{{\upalpha ,42}}}}$$

For the proof see Appendix C in the Supplementary material.

The above implies that if two compartments with different equivalents of non-permeating species are both in equilibrium with another compartment without non-permeating species, then they are also in equilibrium with each other. Furthermore, the G–D factor for two compartments with different equivalents of non-permeating species may be calculated from the G–D factors for these compartments versus the compartment without non-permeating species.

## Special cases

Below we show the calculations of the G–D factor for a couple of special cases of equilibrium between two compartments with multi-ion solutions.

**Case 1** Let us assume that all positive and negative permeating ions have the same absolute charge number (s=2, ${\text{v}}_{{1}} {\text{ = - v}}_{{2}} { > 0}$)*.* Then, given that ${\upgamma }_{{1}} { = 1}$ (by definition), from Eq. (15) we get:

26 $${\text{x}}^{{2}} {{ + \upgamma }}_{{\text{np,2}}} {\text{x - }}\left( {{{1 + \upgamma }}_{{\text{np,1}}} } \right){ = 0}$$

The solution of this equation is:

27 $${\text{x = DF}}_{{1,21}} { = }\frac{{{\text{C}}_{{1,2}} }}{{{\text{C}}_{{1,1}} }}{ = }\frac{{{{ - \upgamma }}_{{\text{np,2}}} { + }\sqrt {{\upgamma }_{{\text{np,2}}}^{{2}} { + 4}\left( {{{1 + \upgamma }}_{{\text{np,1}}} } \right)} }}{{2}}$$

In terms of concentrations Eq. (27) reads:

28 $${\text{DF}}_{{1,21}} { = }\frac{{{\text{ - Z}}_{{\text{np,2}}} {\text{C}}_{{\text{np,2}}} { + }\sqrt {\left( {{\text{Z}}_{{\text{np,2}}} {\text{C}}_{{\text{np,2}}} } \right)^{{2}} {\text{ + 4v}}_{{1}} {\text{C}}_{{1,1}} \left( {{\text{v}}_{{1}} {\text{C}}_{{1,1}} {\text{ + Z}}_{{{\text{np}}{.1}}} {\text{C}}_{{\text{np,1}}} } \right)} }}{{{\text{2v}}_{{1}} {\text{C}}_{{1,1}} }}$$

If the concentrations in compartment 2 are known, then we have the symmetrical expression for concentrations in compartment 1:

29 $$\frac{{{\text{C}}_{{1,1}} }}{{{\text{C}}_{{1,2}} }}{ = }\frac{{1}}{{{\text{DF}}_{{1,21}} }}{ = }\frac{{{\text{ - Z}}_{{\text{np,1}}} {\text{C}}_{{\text{np,1}}} { + }\sqrt {\left( {{\text{Z}}_{{\text{np,1}}} {\text{C}}_{{\text{np,1}}} } \right)^{{2}} {\text{ + 4v}}_{{1}} {\text{C}}_{{1,2}} \left( {{\text{v}}_{{1}} {\text{C}}_{{1,2}} {\text{ + Z}}_{{\text{np,2}}} {\text{C}}_{{\text{np,2}}} } \right)} }}{{{\text{2v}}_{{1}} {\text{C}}_{{1,2}} }}$$

If $${\text{Z}}_{{\text{np,2}}} {\text{C}}_{{\text{np,2}}} { = 0}$$ then:

30 $${\text{DF}}_{{1,21}} { = }\frac{{{\text{C}}_{{1,2}} }}{{{\text{C}}_{{1,1}} }}{ = }\sqrt {{\text{1 + Z}}_{{\text{np,1}}} {\text{C}}_{{\text{np,1}}} {\text{/v}}_{{1}} {\text{C}}_{{1,1}} }$$

For two cations with the charge number + 1 and one anion with the charge number − 1, as for example in a solution of Na^+^, K^+^ and Cl^−^, one can express the G–D factor using the combined concentration of cations K^+^ and Na^+^ (C_1_) as in Eq. (27) or using individual concentrations of each cation separately (c_1_ and c_2_), as follows (derived from an equation analogous to Eq. (15) for individual ions with concentration c_i_ and charge number z_i_, and assuming ${\text{z}}_{{1}} {\text{ = z}}_{{2}} { = 1}$, ${\text{z}}_{{3}} { = - 1}$):

31 $${\text{x = }}\frac{{{{ - \lambda }}_{{\text{np,2}}} { + }\sqrt {{\uplambda }_{{\text{np,2}}}^{{2}} { + 4}\left( {{{1 + \lambda }}_{{2}} } \right)\left( {{{1 + \lambda }}_{{2}} {{ + \lambda }}_{{\text{np,1}}} } \right)} }}{{{2}\left( {{{1 + \lambda }}_{{2}} } \right)}}$$

where $$\lambda_{i} = z_{i} c_{i,1} /\left( {z_{1} c_{1,1} } \right)$$, $${\uplambda }_{{\text{np,1}}} {\text{ = Z}}_{{\text{np,1}}} {\text{C}}_{{\text{np,1}}} {/}\left( {{\text{z}}_{{1}} {\text{c}}_{{1,1}} } \right)$$, and $${\uplambda }_{{\text{np,2}}} {\text{ = Z}}_{{\text{np,2}}} {\text{C}}_{{\text{np,2}}} {/}\left( {{\text{z}}_{{1}} {\text{c}}_{{1,1}} } \right)$$.

**Case 2** Let us consider one bivalent cation and one monovalent anion, as in a solution of Ca^2+^ and Cl^−^. Then, taking the bivalent cation as a reference species (v_1_ = 2), Eq. (15) takes the form:

32 $${\text{x}}^{{3/2}} { - }\left( {{{1 + \upgamma }}_{{\text{np,1}}} } \right){{ + \upgamma }}_{{\text{np,2}}} {\text{x}}^{{1/2}} { = 0}$$

and, denoting $${\text{y = x}}^{{1/2}}$$:

33 $${\text{y}}^{{3}} {{ + \upgamma }}_{{\text{np,2}}} {\text{y - }}\left( {{{1 + \upgamma }}_{{\text{np,1}}} } \right){ = 0}$$

## Equilibrium distribution of permeating ions among compartments

In many real-world problems related to multi-ion solutions separated by a permselective membrane we know the total mass of each permeating ion and the equivalents of non-permeating charged species in each compartment. Regardless of the initial distribution of permeating ions, the electroneutral system tends to an equilibrium described by Eq. (7), with the balance of charge described by Eqs. (8, 9), and the balance of mass for each ion as follows:

34 $${\text{c}}_{{\text{i,1}}} {\text{V}}_{{1}} {\text{ + c}}_{{\text{i,2}}} {\text{V}}_{{2}} {\text{ = M}}_{{\text{i}}}$$

where V denotes the equilibrium volume of the compartment and M_i_ denotes the total mass of the i-th ion. Here we assume that V_1_, V_2_ and M_i_ for all i are known, and the equilibrium concentrations in the two compartments, c_i,1_ and c_i,2_, for all permeating ions need to be found.

For ions with the same charge number ${\text{v}}_{{\upalpha }}$ we have:

35 $${\text{C}}_{{{{\upalpha ,1}}}} {\text{V}}_{{1}} {\text{ + C}}_{{{{\upalpha ,2}}}} {\text{V}}_{{2}} {\text{ = M}}_{{\upalpha }}$$

or, using the G–D factor:

36 $${\text{C}}_{{{{\upalpha ,1}}}} {\text{V}}_{{1}} {\text{ + DF}}_{{{{\upalpha ,21}}}} {\text{C}}_{{{{\upalpha ,1}}}} {\text{V}}_{{2}} {\text{ = M}}_{{\upalpha }}$$

and therefore:

37 $${\text{C}}_{{{{\upalpha ,1}}}} { = }\frac{{{\text{M}}_{{\upalpha }} }}{{{\text{V}}_{{1}} {\text{ + DF}}_{{{{\upalpha ,21}}}} {\text{V}}_{{2}} }}$$

38 $${\text{C}}_{{{{\upalpha ,2}}}} { = }\frac{{{\text{M}}_{{\upalpha }} {\text{ - C}}_{{{{\upalpha ,1}}}} {\text{V}}_{{1}} }}{{{\text{V}}_{{2}} }}$$

In the case of monovalent permeating ions (s = 1) the solution of the above problem is straightforward, given the simple form of ${\text{DF}}_{{{{\upalpha ,21}}}}$ that depends only on the ratio of equivalents of non-permeating species on the two sides of the membrane (see Eq. (22)):

39 $${\text{C}}_{{{{\upalpha ,1}}}} { = }\frac{{{\text{Z}}_{{\text{np,1}}} {\text{C}}_{{\text{np,1}}} {\text{V}}_{{1}} }}{{{\text{Z}}_{{\text{np,1}}} {\text{C}}_{{\text{np,1}}} {\text{V}}_{{1}} {\text{ + Z}}_{{\text{np,2}}} {\text{C}}_{{\text{np,2}}} {\text{V}}_{{2}} }}\frac{{{\text{M}}_{{\upalpha }} }}{{{\text{V}}_{{1}} }}$$

In general, however, the G–D factors depend on the equivalents of all permeating ions and therefore Eq. (37) cannot be used directly to calculate ${\text{C}}_{{{{\upalpha ,1}}}}$. Instead, all equations for mass and charge balance Eqs. (8, 9, 36) need to be solved together. Thus, using Eq. (17), ${\text{DF}}_{{{{\upalpha ,21}}}} {\text{ = x}}^{{{\text{v}}_{{\upalpha }} {\text{/v}}_{{1}} }}$, one gets from Eq. (37):

40 $${\text{C}}_{{{{\upalpha ,1}}}} { = }\frac{{{\text{A}}_{{\upalpha }} }}{{{\text{B + x}}^{{{\text{v}}_{{\upalpha }} {\text{/v}}_{{1}} }} }}$$

where ${\text{A}}_{{\upalpha }} {\text{ = M}}_{{\upalpha }} {\text{/V}}_{{2}}$ is an apparent concentration of ions with the charge number ${\text{v}}_{{\upalpha }}$ if their total amount was distributed in compartment 2, and ${\text{B = V}}_{{1}} {\text{/V}}_{{2}}$. Then, the coefficients γ in Eq. (11) may be transformed to:

41 $${{ \upgamma }}_{{\upalpha }} { = }\frac{{{\text{v}}_{{\upalpha }} {\text{A}}_{{\upalpha }} }}{{{\text{v}}_{{1}} {\text{A}}_{{1}} }}\frac{{\text{B + x}}}{{{\text{B + x}}^{{{\text{v}}_{{\upalpha }} {\text{/v}}_{{1}} }} }}$$

and:

42 $${\upgamma }_{{\text{np,2}}} { = }\frac{{{\text{Z}}_{{\text{np,2}}} {\text{C}}_{{\text{np,2}}} }}{{{\text{v}}_{{1}} {\text{A}}_{{1}} }}\left( {\text{B + x}} \right)$$

Therefore, Eq. (11) transforms to:

43 $$\left( {\text{B + x}} \right)\left( {\sum\limits_{{{{\upalpha = 1}}}}^{{\text{s}}} {\frac{{{\text{v}}_{{\upalpha }} {\text{A}}_{{\upalpha }} }}{{{\text{v}}_{{1}} {\text{A}}_{{1}} }}\frac{{{\text{x}}^{{{\text{v}}_{{\upalpha }} {\text{/v}}_{{1}} }} }}{{{\text{B + x}}^{{{\text{v}}_{{\upalpha }} {\text{/v}}_{{1}} }} }}} { + }\frac{{{\text{Z}}_{{\text{np,2}}} {\text{C}}_{{\text{np,2}}} }}{{{\text{v}}_{{1}} {\text{A}}_{{1}} }}} \right){ = 0}$$

and finally:

44 $$\sum\limits_{{{{\upalpha = 1}}}}^{{\text{s}}} {\upphi_{{\upalpha }} \frac{{{\text{x}}^{{{\text{v}}_{{\upalpha }} {\text{/v}}_{{1}} }} }}{{{\text{B + x}}^{{{\text{v}}_{{\upalpha }} {\text{/v}}_{{1}} }} }}} { + }\upphi_{{\text{np,2}}} { = 0}$$

where $\upphi_{\upalpha } = v_{\upalpha } A_{\upalpha } /\left( {v_{1} A_{1} } \right)$ and $\upphi_{{\text{np,2}}} {\text{ = Z}}_{{\text{np,2}}} {\text{C}}_{{\text{np,2}}} {/}\left( {{\text{v}}_{{1}} {\text{A}}_{{1}} } \right)$ are apparent relative ionic equivalents for the distribution of all permeating ions in compartment 2.

In general, Eq. (44) needs to be solved numerically, and once the G–D factors are found, the equilibrium concentrations may be calculated using Eq. (40).

Equation (44) may be also presented in other forms, as for example the one analogous to Eq. (16) with $y = x^{{1/v_{1} }}$:

45 $$\sum\limits_{{{{\upalpha = 1}}}}^{{\text{s - 1}}} {\upphi_{{\upalpha }} \frac{{{\text{y}}^{{{\text{v}}_{{{\text{max}}}} {\text{ + v}}_{{\upalpha }} }} }}{{{\text{B + y}}^{{{\text{v}}_{{\upalpha }} }} }}} { - }\left( {\sum\limits_{{{{\upalpha = 1}}}}^{{\text{s - 1}}} {\frac{{\upphi_{{\upalpha }} }}{{{\text{B + y}}^{{{\text{v}}_{{\upalpha }} }} }}} { + }\upphi_{{\text{np,1}}} } \right){\text{y}}^{{{\text{v}}_{{{\text{max}}}} {\text{ + v}}_{{\text{s}}} }} { + }\upphi_{{\text{np,2}}} {\text{y}}^{{{\text{v}}_{{{\text{max}}}} }} { = 0}$$

where $$\upphi_{{\text{np,1}}} {\text{ = Z}}_{{\text{np,1}}} {\text{C}}_{{\text{np,1}}} {/}\left( {{\text{v}}_{{1}} {\text{A}}_{{1}} } \right)$$.

In the case $s = 1$ one has ${\text{x = }}\frac{{\upphi_{{\text{np,2}}} }}{{\upphi_{{\text{np,1}}} }}$.

In the special case of the same absolute charge number of positive and negative permeating ions, i.e. ${\text{s = 2}}$, ${\text{v}}_{{1}} {\text{ = - v}}_{{2}} { > 0}$, as for example in a solution of Na^+^ and Cl^−^ (${\text{v}}_{{1}} { = 1,}\;{\text{v}}_{{2}} { = - 1}$), Eq. (45) can be reduced to:

46 $$\frac{{{\text{x}}^{{2}} { - 1}}}{{\text{B + x}}}{ + }\upphi_{{\text{np,2}}} {\text{x - }}\upphi_{{\text{np,1}}} { = 0}$$

and:

47 $$\left( {{1 + }\upphi_{{\text{np,2}}} } \right){\text{x}}^{{2}} { - }\left( {\upphi_{{\text{np,1}}} { - }\upphi_{{\text{np,2}}} {\text{B}}} \right){\text{x - }}\left( {{1 + }\upphi_{{\text{np,1}}} {\text{B}}} \right){ = 0}$$

with the solution:

48 $${\text{x = }}\frac{{\left( {\upphi_{{\text{np,1}}} { - }\upphi_{{\text{np,2}}} {\text{B}}} \right){{ \pm }}\sqrt {\left( {\upphi_{{\text{np,1}}} { - }\upphi_{{\text{np,2}}} {\text{B}}} \right)^{{2}} { + 4}\left( {{1 + }\upphi_{{\text{np,2}}} } \right)\left( {{1 + }\upphi_{{\text{np,1}}} {\text{B}}} \right)} }}{{{2}\left( {{1 + }\upphi_{{\text{np,2}}} } \right)}}$$

For another case of ${\text{s = 2}}$ and ${\text{v}}_{{1}} { = 2,}\,\,{\text{v}}_{{2}} { = - 1}$, as in a solution of Ca^+^ and Cl^−^:

49 $$\frac{{{\text{y}}^{{4}} {\text{ - y}}}}{{{\text{B + y}}^{{2}} }}{ + }\upphi_{{\text{np,2}}} {\text{y}}^{{2}} { - }\upphi_{{\text{np,1}}} {\text{y = 0}}$$

This equation requires numerical solution.

## Examples

### Example 1 Two compartments, two monovalent permeating ions, non-permeating charge in one compartment only (a classic example of the Gibbs–Donnan equilibrium)

Let us consider the distribution of two main ions in human blood plasma and interstitial fluid, Na^+^ and Cl^−^, with the two fluids being separated by permselective capillary walls. Let us also assume that the non-permeating charged species (proteins) are present only in plasma (compartment 1) with the equivalent ${\text{Z}}_{{\text{np,1}}} {\text{C}}_{{\text{np,1}}}$, and that there are no other ions in the two solutions. Then, from Eq. (27), the G–D factor for sodium is given by:

50 $${\text{DF}}_{{1,21}} { = }\frac{{{\text{c}}_{{\text{Na,2}}} }}{{{\text{c}}_{{\text{Na,1}}} }}{ = }\sqrt {{{1 + \upgamma }}_{{\text{np,1}}} }$$

where $${\upgamma }_{{\text{np,1}}} {\text{ = Z}}_{{\text{np,1}}} {\text{C}}_{{\text{np,1}}} {\text{/c}}_{{\text{Na,1}}}$$.

Then, for example, if we know that in equilibrium ${\text{c}}_{{\text{Na,1}}} { = 140}$ mmol/L, $c_{Cl,1} = 124$ mmol/L, and ${\text{Z}}_{{{\text{np1}}}} {\text{C}}_{{{\text{np1}}}} { = - 16}$ mEq/L, we have $\frac{{{\text{c}}_{{\text{Na,2}}} }}{{{\text{c}}_{{\text{Na,1}}} }}{ = 0}{\text{.941}}$, $\frac{{{\text{c}}_{{\text{Cl,2}}} }}{{{\text{c}}_{{\text{Cl,1}}} }}{ = 1}{\text{.063}}$, and the concentrations in the interstitial fluid (compartment 2) are ${\text{c}}_{{\text{Na,2}}} {\text{ = c}}_{{\text{Cl,2}}} { = 131}{\text{.8}}$ mmol/L.

Let us assume now (ignoring the physiology) that the non-permeating proteins are present in compartment 2 instead of compartment 1. Then, from Eq. (27):

51 $$\frac{{{\text{c}}_{{\text{Na,2}}} }}{{{\text{c}}_{{\text{Na,1}}} }}{ = }\frac{{{{ - \upgamma }}_{{\text{np,2}}} { + }\sqrt {{\upgamma }_{{\text{np,2}}}^{{2}} { + 4}} }}{{2}}$$

Assuming, for example, that in equilibrium ${\text{c}}_{{\text{Na,1}}} {\text{ = c}}_{{\text{Cl,1}}} { = 140}$ mmol/L, and ${\text{Z}}_{{{\text{np2}}}} {\text{C}}_{{{\text{np2}}}} { = - 16}$ mEq/L, we have $\frac{{{\text{c}}_{{\text{Na,2}}} }}{{{\text{c}}_{{\text{Na,1}}} }}{ = 1}{\text{.059}}$, $\frac{{{\text{c}}_{{\text{Cl,2}}} }}{{{\text{c}}_{{\text{Cl,1}}} }}{ = 0}{\text{.944}}$, and the concentrations in compartment 2 are ${\text{c}}_{{\text{Na,2}}} { = 148}{\text{.2}}$ mmol/L, ${\text{c}}_{{\text{Cl,2}}} { = 132}{\text{.2}}$ mmol/L.

### Example 2 Three compartments, two monovalent permeating ions, non-permeating charge in two compartments

Let us consider again a simplified case of human blood plasma separated by permselective capillary walls from the interstitial fluid and a dialysis fluid separated from plasma by a permselective dialyzer membrane, with all fluids containing two monovalent permeating ions (Na^+^ and Cl^−^). Let us also assume that the non-permeating charged species (proteins) are present in plasma (compartment 1; equivalent Z_np,1_C_np,1_ = − 16 mEq/L) and in the interstitial fluid (compartment 2; equivalent Z_np,2_C_np,2_ = − 8 mEq/L), whereas the dialysis fluid (compartment 3) contains only the permeating ions. If we know the equilibrium ion concentrations in compartment 3, for example ${\text{c}}_{{\text{Na,3}}} {\text{ = c}}_{{\text{Cl,3}}} { = 140}$ mmol/L, then the equilibrium concentrations in compartment 1 are ${\text{c}}_{{\text{Na,1}}} { = 148}{\text{.2}}$ mmol/L, ${\text{c}}_{{\text{Cl,1}}} { = 132}{\text{.2}}$ mmol/L, and in compartment 2: ${\text{c}}_{{\text{Na,2}}} { = 144}{\text{.1}}$ mmol/L, ${\text{c}}_{{\text{Cl,2}}} { = 136}{\text{.1}}$ mmol/L (calculated similarly as in the second part of the previous example). The corresponding G–D factors are therefore $\frac{{{\text{c}}_{{\text{Na,2}}} }}{{{\text{c}}_{{\text{Na,1}}} }}{ = 0}{\text{.972}}$ and $\frac{{{\text{c}}_{{\text{Cl,2}}} }}{{{\text{c}}_{{\text{Cl,1}}} }}{ = 1}{\text{.029}}$, which can be also estimated directly using Eq. (28), based on the known protein equivalents in compartments 1 and 2, and known concentrations of Na^+^ and Cl^−^ in compartment 1.

### Example 3 Two compartments, two monovalent permeating ions, non-permeating charge in both compartments

**Figure 4:**
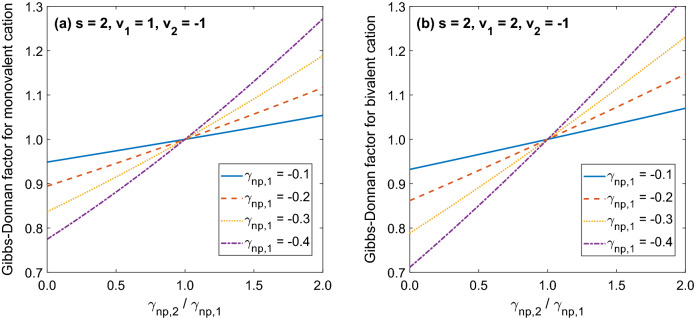
The relationship between the Gibbs–Donnan factor for the reference permeating monovalent (left panel) or bivalent (right panel) cation (for example Na^+^ or Ca^2+^ in a solution with Cl^−^) and the ratio ${\upgamma }_{{\text{np,2}}} / \upgamma _{{\text{np,1}}} = Z_{{\text{np,2}}} {\text{C}}_{{\text{np,2}}}/ {\text{Z}}_{{\text{np,1}}} {\text{C}}_{{\text{np,1}}}$ of equivalents of non-permeating species on the two sides of a permselective membrane, calculated according to Eq. (15) for different values of γ_np,1_ (the case with known ion concentrations in compartment 1).

As is clear from Eq. (15) and from the previous examples, if we know the equilibrium concentrations of permeating ions on one side of a permselective membrane, then their concentrations on the other side of the membrane, or their G–D factors, depend on the equivalents of non-permeating charged species on the two sides of the membrane. Figure 4 shows how the G–D factor depends on the ratio of the equivalents of non-permeating species on the two sides of the membrane (${\upgamma }_{{\text{np,2}}} {{/ \upgamma }}_{{\text{np,1}}} {\text{ = Z}}_{{\text{np,2}}} {\text{C}}_{{\text{np,2}}} {\text{/Z}}_{{\text{np,1}}} {\text{C}}_{{\text{np,1}}}$) for two special cases discussed earlier, i.e. a solution of Na^+^ and Cl^−^ (Eq. (27)) and a solution of Ca^2+^ and Cl^−^ (Eq. (33)), for different levels of the relative equivalent of non-permeating species in compartment 1 (γ_np,1_). Note that for the same equivalent of non-permeating species and with the ratio ${\upgamma }_{{\text{np,2}}} {{/ \upgamma }}_{{\text{np,1}}}$ in the range 0–1, the G–D factor for a bivalent cation (Ca^2+^ in the solution with Cl^−^) is always lower than that of a monovalent cation (Na^+^ in the solution with Cl^−^).

**Figure 5:**
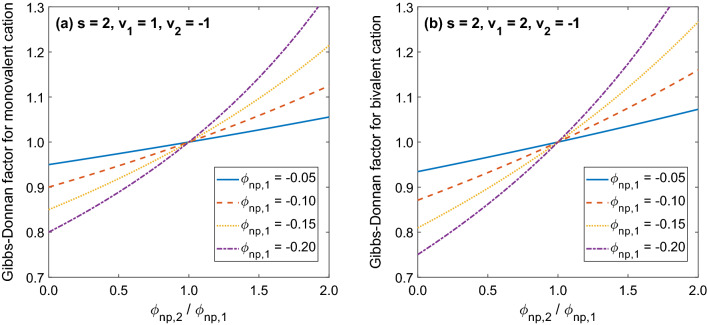
The relationship between the Gibbs–Donnan factor for the reference permeating monovalent (left panel) or bivalent (right panel) cation (for example Na^+^ or Ca^2+^ in a solution with Cl ^−^) and the ratio $\upphi_{{\text{np,2}}} {/}\upphi_{{\text{np,1}}} {\text{ = Z}}_{{\text{np,2}}} {\text{C}}_{{\text{np,2}}} {\text{/Z}}_{{\text{np,1}}} {\text{C}}_{{\text{np,1}}}$ of equivalents of non-permeating species on the two sides of a permselective membrane, calculated according to Eq. (45) for different values of ϕ_np,1_ (the case with known total amounts of permeating ions distributed among the two compartments of the same volume, i.e. B = 1).

Figure 5 shows a similar relationship as Fig. 4 but for the case when, instead of knowing the equilibrium concentrations of permeating ions on one side of the membrane, we know the total amounts of permeating ions distributed among the two compartments (Eq. (45)) assuming the same volume of both compartments at equilibrium (B = 1). Note that in this case the equivalent of non-permeating species in compartment 1 is expressed in relation to the equivalent of the reference permeating ion in both compartments (φ_np,1_), and hence, for small values, φ_np,1_ in Fig. 5 corresponds roughly to γ_np,1_ = 2φ_np,1_ in Fig. 4.

### Example 4 Blood plasma and interstitial fluid separated by a capillary wall

Here, we consider a more detailed description of the human blood plasma and interstitial fluid that are in contact in the capillary beds of various body tissues, with the capillary walls acting as permselective membranes enabling the exchange of certain solutes between blood and tissues, while keeping some other solutes and blood cells within the vasculature. Both fluids contain multiple small permeating ions as well as proteins, which, for simplicity, are treated here as non-permeating charged species, even though some of them (e.g. albumin and small globulins) are actually able to pass through the capillary walls, and hence under physiological conditions (even in a steady state) there is a continuous flow of proteins from plasma to the interstitial fluid.

Exemplary concentrations of main small ions in human plasma and interstitial fluid (expressed in osmoles per liter of water) are shown in Table 1. ^17^. Assuming that these nine ions along with the negatively charged, non-permeating proteins constitute most of the charged species in plasma and interstitial fluid, one may calculate the equivalent of proteins using the condition of electroneutrality of the two fluids as follows: the protein equivalent in plasma − 12.2 mEq/L and in the interstitial fluid − 4.3 mEq/L (for simplicity, we ignored here the fact that proteins have a non-negligible volume, and hence the ion concentrations in plasma or interstitial fluid differ from their concentrations in the water fractions of these fluids reported in Table 1). Using these protein equivalents and the equilibrium concentrations of small permeating ions in plasma (where they are typically measured) we have calculated the G–D factors for all permeating ions and their equilibrium concentrations in the interstitial fluid using Eqs. (16, 18), see Table 1. Note that the empirical concentrations provided in Table 1 do not come from any systematic measurements but are rather examples of typical values seen in the general population, which may explain some discrepancy between the empirical and theoretical (estimated) ion concentrations in the interstitial fluid. This discrepancy can be further explained by the aforementioned disregard of water fractions of the considered fluids, the somewhat limited number of ions considered, as well as by the fact that both plasma and interstitial fluid are non-ideal solutions in which some of the ions form various species with their counterions or are bound to proteins (therefore, the empirical concentrations provided in Table 1 are not necessarily the activities of free ion fractions, as considered in the presented theory). We also ignored here the fact that on the tissue side of the capillary walls there are some non-permeating negative charges other than proteins (fixed negative charges of the extracellular matrix) that affect the G–D effect and the electroneutrality conditions. Moreover, the provided empirical concentrations may not correspond to a static equilibrium, since physiological fluids are often in dynamic equilibrium. The largest difference can be observed for bicarbonate ions (HCO3^−^), which constitute the base buffer, and hence are subject to various chemical reactions not considered in the theory presented here.

**Table 1:** Empirical equilibrium concentrations of main small ions (in mOsm/L H_2_O) in human blood plasma (assumed total protein equivalent: − 12.2 mEq/L) and interstitial fluid (assumed total protein equivalent: − 4.3 mEq/L) from Guyton and Hall^17^ and the associated equilibrium concentrations of ions in the interstitial fluid along with the theoretical values of the Gibbs–Donnan factor calculated using the presented method (Eq. (16)).

Ion	Empirical equilibrium concentrations: plasma	Empirical equilibrium concentrations: interstitial fluid	Estimated equilibrium concentrations: interstitial fluid	Estimated Gibbs–Donnan factor
Na^+^	142.0	139.0	138.3	0.97
K^+^	4.2	4.0	4.1	0.97
Ca^2+^	1.3	1.2	1.2	0.95
Mg^2+^	0.8	0.7	0.8	0.95
Cl^−^	108.0	108.0	110.9	1.03
HCO_3_^−^	24.0	28.3	24.7	1.03
Lactate^−^	1.2	1.2	1.2	1.03
SO_4_^2−^	0.5	0.5	0.5	1.06
HPO_4_^2−^	2.0	2.0	2.1	1.06

In the same way we calculated the G–D factors of small permeating ions in the two considered fluids versus a protein-free solution (assuming that such a solution would be present on the other side of a permselective membrane)—see Table 2 for plasma and Table 3 for interstitial fluid. The transitivity of the G–D factors (Eq. (25)) can be now easily checked based on the G–D factors provided in Tables 1, 2, 3.

**Table 2:** Empirical equilibrium concentrations of main small ions (in mOsm/L H_2_O) in human blood plasma (assumed protein equivalent: − 12.2 mEq/L) from Guyton and Hall^17^ and the associated equilibrium concentrations of ions in a protein-free fluid along with the theoretical values of the Gibbs–Donnan factor calculated using the presented method (eq. (16)).

Ion	Empirical equilibrium concentrations: plasma	Estimated equilibrium concentrations: protein-free fluid	Estimated Gibbs–Donnan factor
Na^+^	142.0	136.3	0.96
K^+^	4.2	4.0	0.96
Ca^2+^	1.3	1.2	0.92
Mg^2+^	0.8	0.7	0.92
Cl^−^	108.0	112.5	1.04
HCO_3_^−^	24.0	25.0	1.04
Lactate^−^	1.2	1.3	1.04
SO_4_^2−^	0.5	0.5	1.09
HPO_4_^2−^	2.0	2.2	1.09

**Table 3:** Estimated equilibrium concentrations of main small ions (in mOsm/L H_2_O) in human interstitial fluid (assumed protein equivalent: − 4.3 mEq/L) taken from Table 1 and the equilibrium concentrations of ions in a protein-free fluid along with the theoretical values of the Gibbs–Donnan factor calculated using the presented method (eq. (16)).

Ion	Estimated equilibrium concentrations: interstitial fluid	Estimated equilibrium concentrations: protein-free fluid	Estimated Gibbs–Donnan factor
Na^+^	138.3	136.3	0.99
K^+^	4.1	4.0	0.99
Ca^2+^	1.2	1.2	0.97
Mg^2+^	0.8	0.7	0.97
Cl^−^	110.9	112.5	1.02
HCO_3_^−^	24.7	25.0	1.02
Lactate^−^	1.2	1.3	1.02
SO_4_^2−^	0.5	0.5	1.03
HPO_4_^2−^	2.1	2.2	1.03

Note that in all three tables we present the G–D factors with two decimals only. Given that the G–D factor can be calculated directly from the known concentrations of any individual ion in the two considered compartments (DF_i,21_ = c_i,2_/c_i,1_), according to the theory of error propagation and assuming c_i,1_ ≈ c_i,2_ = c (as observed in the considered physiological example), the expected error of the G–D factor may be approximated as 2Δc/c, where Δc is the error of measurement of ion concentration. Assuming that the G–D factor was calculated from the concentrations of sodium (the most abundant ion in human blood plasma) measured with the accuracy of 0.1 mmol/L (as provided in Tables 1 and 2), the expected error amounts to 0.0014, which is why we used the conservative level of accuracy of 0.01.

In the case considered here, given that there are four different charge numbers of permeating ions, the Eq. (16) requires a numerical solution. The Excel-based calculator provided as a supplementary material enables the calculation of the G–D factors for such complex cases. The calculator includes a predefined (editable) example with the present case describing the equilibrium of main ions seen in human plasma and interstitial fluid. The user may freely change the composition of both fluids (including their water fractions ignored above) to see how it affects the calculated G–D factors and equilibrium ion concentrations. The calculator can also be used for the calculation of the G–D factors for other multi-ion solutions with various equivalents of non-permeating species in the two compartments separated by a permselective membrane.

## Discussion

In mixtures of ions separated by a permselective membrane that is permeable to some of them (permeating ions) and not permeable to other (non-permeating charged species), the G–D equilibrium depends on the ionic equivalents of all ions present in the mixture on one or both sides of the membrane. However, in many studies, particularly in physiology and medicine, the G–D equilibrium is often assumed for each permeating ion as if it was the only ion in the solution, and as if the non-permeating charges were present on one side of the membrane only, even if they are actually present on both sides. Yet, even for such complex ideal solutions the equilibrium ion concentrations can be calculated using relatively simple mathematics. Namely, we showed that the problem of finding the G–D equilibrium for such ideal multi-ion mixtures can be solved using a polynomial equation for the concentration ratio of one (arbitrarily selected) permeating ion on the two sides of the membrane or one group of permeating ions with the same charge number, as demonstrated by Eq. (16). This ratio, called the G–D factor, is a function of the equivalents (i.e. products of molar concentration and charge number) of all permeating ions on one side of the membrane and the equivalents of non-permeating species on both sides of the membrane. Assuming that these equivalents are known, one can calculate the concentration ratio for the selected ion using Eq. (16) and then for other ions using Eq. (18). In some cases, Eq. (16) can be reduced to the second order polynomial, as for the monovalent ions, Eq. (26), and then it may be solved analytically. If the mixture includes both monovalent and bivalent ions, then the equation is of the third order, see Eq. (33). Finally, the unknown equilibrium concentrations of all permeating ions on the other side of the membrane may be calculated.

In the presented equations and examples, we assumed that the considered ion mixtures are ideal solutions (with ion activities equal to ion concentrations and independent from other ion concentrations) and that there are no chemical compounds (pairs or complexes) formed by individual ions and their counterions. In the Supplementary material (Appendix A and B) we provide a generalized version of the G–D theory for non-ideal solutions (with ion activities depending on the concentrations of all ions in the mixture), in which ions may form various species with their counterions. This may be particularly important for multivalent ions, for which ion activities may strongly deviate from ion concentrations. In such cases, the solutions of the equations become, however, much more complex.

Typical applications of the G–D theory include permselective membranes separating multi-ion solutions with non-permeating, charged macromolecules, as is often the case with biological fluids studied in physiology, microbiology, food industry, and wastewater treatment. Within the field of physiology and medicine, the G–D effect applies to fluid and solute exchange across the capillary walls (microvascular exchange)^22^, across the cellular membranes (in this case, active transport across the membrane needs to be considered for some ions, such as sodium or potassium^11^), or across extracorporeal membranes, such as during hemodialysis^23^. In particular, the G–D effect is crucial when considering osmotic equilibria between the intra- and extracellular fluids as well as between the intra- and extravascular fluids^24,25^, e.g. for the analysis of cellular or tissue edema^16,26^.

We showed that the G–D factors have an important feature of transitivity: if we know the standard G–D factors of two solutions with different equivalents of non-permeating macromolecules against a macromolecule-free solution, then the G–D factors that describe the equilibrium between the two solutions with macromolecules can be calculated from those standard G–D factors using Eqs. (24, 25), as can be seen in Example 4.

Another important feature of the presented formulas is the additivity of the concentrations (or activities) of all ions with the same charge number, i.e. to calculate the G–D factors one may use the sum of concentrations of all ions with the same charge number instead of considering all ions separately.

Note that in our equations we use a certain (arbitrarily selected) order of permeating ions (concentrations C_1_, C_2_,…C_s_ of ions or groups of ions with charge numbers v_1_,v_2_,…v_s_), with the first ion, or group of ions, treated as a reference species for which the G–D factor (x) is calculated using Eqs. (16, 45). If one changes the order of ions, in particular if one changes the reference species, the obtained equations will be different polynomials but the ultimate global solution for the multi-ion mixture will remain the same, i.e. regardless of the applied ion order, the G–D factors for individual ions and their equilibrium concentrations will be the same.

From a practical point of view, when discussing the G–D factors one should take into account the incomplete dissociation of salts into ions and the potential binding of ions to macromolecules (the G–D factor should be applied only to free ions^27^). Depending on the case, the role of pH needs also to be critically evaluated, especially for proteins and buffer ions. One should also remember that the concentrations of ions are often measured per volume of the whole fluid, whereas their distribution is solvent (e.g. water); therefore, a correction for the volume of macromolecules (or solvent/water fraction of the fluid) may be necessary^21^. Moreover, some experiments may need a correction for absorption of ions and their accumulation inside the membrane, or, especially in long experiments, for non-ideal semi-permeability of the membrane that might be somewhat leaky to some of the ions treated as non-permeating. It is worth noting, however, that our method assumes constant equivalents of non-permeating species on the two sides of the membrane, and hence such a leak may still be acceptable, provided that some other mechanisms keep those equivalents constant on each side of the membrane (as for example the lymphatic drainage of interstitial fluid that keeps plasma and interstitial protein levels relatively constant in normal conditions despite the natural leakage of some proteins through the capillary walls). On the other hand, by considering total equivalents of non-permeating species, we ignore the fact that the individual non-permeating species may have a largely different charge per molecule (even of opposite sign), which could affect, in a non-linear way, ion binding (not considered here). In particular, in the case of blood plasma and interstitial fluid, different types of proteins (albumin and various globulins) not only have a different net molecular charge (depending on the pH level) but have many isolated charged residues on their surface, thus affecting local ion binding. Finally, note that the G–D factors for small ions in biological fluids are relatively close to unity but their precise value may be of importance for ions whose concentrations are similar on both sides of the membrane, as for example in sodium and calcium transport in hemodialysis or peritoneal dialysis^21,27^.

## Conclusions

In this study we presented a comprehensive method for calculating the Gibbs–Donnan factors for ideal electroneutral multi-ion solutions separated by a permselective membrane for cases when non-permeating charged species are present on both sides of the membrane, such as the case of capillary walls separating blood plasma and interstitial fluid with different amounts of negatively charged proteins in the two fluids.

The presented equations for the Gibbs–Donnan equilibrium may be used both when one needs to calculate ion concentrations in one compartment based on their concentrations in the other compartment or when one wants to calculate the equilibrium concentrations in the two compartments based on the total amount of ions to be distributed among them.

## Supplementary Information

Supplementary Information 1.

Supplementary Information 2.

## Data Availability

No datasets were generated or analyzed during the current study.
