# Supplementary material for: Calculation of the Gibbs–Donnan factors for multi-ion solutions with non-permeating charge on both sides of a permselective membrane
Source: Sci Rep. 2021 Nov 12;11:22150. doi: 10.1038/s41598-021-00899-y (PMC8589849; doi:10.1038/s41598-021-00899-y)
Supplement: Supplementary file 2 — Supplementary Information 2. [file 41598_2021_899_MOESM2_ESM.pdf]

Jacek Waniewski, Mauro Pietribiasi, Leszek Pstras

"Calculation of the Gibbs–Donnan factors for multi-ion solutions with non-permeating charge on both sides of a permselective membrane"

Appendix A. Calculation of the Gibbs–Donnan factors for non-ideal solutions

From equation (4), the activities of any two ions with charge numbers z_i and z_j (i, j = 1,2,...n) in the two considered compartments can be related as follows:

$$\left(\frac{a_{i,2}}{a_{i,1}}\right)^{1/z_i} = \left(\frac{a_{j,2}}{a_{j,1}}\right)^{1/z_j} \tag{A1}$$

In non-ideal multi-ion solutions, the ion activity is a function of the ion concentration as well as concentrations of other ions in the solution: $a_i = f_i\left(c_1, c_2, ..., c_n, C_{np}\right)$. As before, we assume here that the ion concentrations (c_i) represent free (unbound) ion fractions. Let us also assume that the function f_i is invertible, i.e., if one knows the activities of all ions, then their concentrations can be calculated as follows: $c_i = f_i^{-1}\left(a_1, a_2, ..., a_n, A_{np}\right)$. For non-permeating species we assume the analogous functions f_{np} and f_{np}^{-1}.

Thus, using equation (A1), we have:

$$c_{i,2} = f_i^{-1}\left(a_{1,2}, a_{2,2}, ..., a_{n,2}, A_{np,2}\right) = f_i^{-1}\left(x^{z_1/z_1}a_{1,1}, x^{z_2/z_1}a_{2,1}, ..., x^{z_n/z_1}a_{n,1}, A_{np,2}\right) \tag{A2}$$

where $x = a_{1,2} / a_{1,1}$.

From equations (2) and (A2) we have:

$$\sum_{i=1}^{n} z_i f_i^{-1}\left(x^{z_1/z_1}a_{1,1}, ..., x^{z_n/z_1}a_{n,1}, A_{np,2}\right) + Z_{np,2} f_{np}^{-1}\left(x^{z_1/z_1}a_{1,1}, ..., x^{z_n/z_1}a_{n,1}, A_{np,2}\right) = 0 \tag{A3}$$

To calculate the G–D factor for ions with the charge number z_i ($DF_{i,21} = x^{z_i/z_1}$) one needs to solve the above equation for x based on the known activities of permeating ions in compartment 1 ($a_{i,1}$) and non-permeating species in compartment 2 ($A_{np,2}$), assuming that the functions f_i^{-1} and f_{np}^{-1} are known.

Let us assume that we can express ion activity as:

$$a_i = \gamma_i c_i \tag{A4}$$

where the activity coefficient γ_i is a function of concentrations of all ions (not to be confused with the previously defined γ_α describing the relative ionic equivalents).

The Debye-Hückel theory (validated for low ion concentrations) provides the following description of activity coefficient:

$$\gamma_i = \exp\left(-g_i(I)\right) \tag{A5}$$

where:

$$g_i(I) = \frac{A z_i^2 \sqrt{I}}{1 + B r_i \sqrt{I}} \tag{A6}$$

$$I = \frac{1}{2}\sum_{i=1}^{n} z_i^2 c_i \tag{A7}$$

where r_i is the effective ion radius in the solution, A and B are known constants that depend only on the solvent and temperature (not to be confused with A_α and B introduced when describing the equilibrium distribution of permeating ions among two compartments), and I is the so-called ionic strength of the solution.

From equations (A4) and (A5) one has:

$$c_i = \exp\left(g_i(I)\right) a_i \tag{A8}$$

and from equation (A7):

$$I = \frac{1}{2}\left(\sum_{i=1}^{n} z_i^2 \exp\left(g_i(I)\right) a_i + Z_{np}^2 \exp\left(g_i(I)\right) A_{np}\right) \tag{A9}$$

Equation (A9) needs to be solved numerically for I with the known set of all ion activities a_i to get the function I=$\tilde{I}\left(a_1, a_2, ..., a_n, A_{np}\right)$ and finally the formula:

$$c_i = \exp\left(g_i\left(\tilde{I}\left(a_1, a_2, ..., a_n, A_{np}\right)\right)\right) a_i \tag{A10}$$

and so:

$$f_i^{-1}\left(a_1, a_2, ..., a_n, A_{np}\right) = \exp\left(g_i\left(\tilde{I}\left(a_1, a_2, ..., a_n, A_{np}\right)\right)\right) a_i \tag{A11}$$

As is clear from the above, the functions f_i^{-1} and f_{np}^{-1} are highly complex, and hence solving equation (A3) for a multi-ion solution is a very computationally demanding task. Note, however, that in the equation (A1) we have the ratios of ion activities in the two considered compartments, and hence, based on the Debye-Hückel theory, if the ionic strength I and the effective ionic radii r_i are not much different in the two solutions (as may be the case), the activity coefficients γ_i will be similar in both compartments, and therefore the activity ratio $a_{i,2} / a_{i,1}$ could be approximated by the concentration ratio $c_{i,2} / c_{i,1}$.

Appendix B. Mass balance with ion speciation

Regardless of the initial distribution of permeating ions, an electroneutral two-compartment system tends to an equilibrium described by equations (1) and (2), and by the balance of mass for each ion i:

$$c_{i,1}V_1 + c_{i,2}V_2 + \sum_{k:\text{counterions}} \left(c_{ik,1}V_1 + c_{ik,2}V_2\right) = M_i \tag{B1}$$

where V_i denotes the volume of the i-th compartment and M_i denotes the total mass of the i-th ion, c_{ik} is the concentration of the chemical compound built of ion i and its counterion k, the summation is over all counterions to ion i. Here, we assume that V_1, V_2 and M_i for all i are known, and that the equilibrium concentrations of all permeating ions in the two compartments, $c_{i,1}$ and $c_{i,2}$, need to be found.

Assuming the reversibility of the reaction between an ion i and its counterion k that forms a species ik, one has:

$${a_{ik}}^{\nu_{ik}} = K_{ik}{a_i}^{\nu_i}{a_k}^{\nu_k} \tag{B2}$$

where ν are stoichiometric coefficients of the species in the reaction and K_{ik} is the equilibrium constant for the reaction,

$$a_i = f_i\left(c\right) \tag{B3}$$

$$a_{ik} = f_{ik}\left(c\right) \tag{B4}$$

where c denotes the set of all concentrations of permeating and non-permeating species and all ionic compounds $c = \left\{\left\{c_i\right\}, C_{np}, \left\{c_{ik}\right\}\right\}$. $c_i = f_i^{-1}\left(a\right)$ where $a = \left\{\left\{a_i\right\}, A_{np}, \left\{a_{ik}\right\}\right\}$.

For simplicity, we omit here the reactions that can involve three or more different ions and non-reversible reactions, such as precipitation. Thus:

$$c_{ik} = f_{ik}^{-1}\left(\left\{a_i\right\}, A_{np}, \left\{\left(K_{ik}{a_i}^{\nu_i}{a_k}^{\nu_k}\right)^{1/\nu_{ik}}\right\}\right) \tag{B5}$$

The equations (B1), (B5), and (A3) need to be solved together to obtain the G–D factors for the generalized, non-ideal case of multi-ion solutions with various chemical forms of permeating ions i.

Appendix C. Proof of the transitivity of Gibbs–Donnan factors

To prove the transitivity of the Gibbs–Donnan factors, equations (24) and (25), let us consider the charge balance for each compartment in equilibrium:

$$\sum_{\alpha=1}^{s} v_\alpha C_{\alpha,1} + Z_{np,1} C_{np,1} = 0 \tag{C1}$$

$$\sum_{\alpha=1}^{s} v_\alpha C_{\alpha,2} + Z_{np,2} C_{np,2} = 0 \tag{C2}$$

$$\sum_{\alpha=1}^{s} v_\alpha C_{\alpha,3} = 0 \tag{C3}$$

$$\sum_{\alpha=1}^{s} v_\alpha C_{\alpha,4} = 0 \tag{C4}$$

Then, from equations (C3) and (23):

$$\sum_{\alpha=1}^{s} v_\alpha x_{31}^{v_\alpha/v_1} C_{\alpha,1} = 0 \tag{C5}$$

and, from equations (C4) and (23):

$$\sum_{\alpha=1}^{s} v_\alpha x_{42}^{v_\alpha/v_1} x_{21}^{v_\alpha/v_1} C_{\alpha,1} = 0 \tag{C6}$$

The difference of the last two equations yields:

$$\sum_{\alpha=1}^{s} v_\alpha \left(x_{31}^{v_\alpha/v_1} - x_{42}^{v_\alpha/v_1} x_{21}^{v_\alpha/v_1} \right) C_{\alpha,1} = 0 \tag{C7}$$

The separation of the sum in equation (C7) into two terms that include m ions with a positive charge number and s-m ions with a negative charge number yields the following (here we assume, for the sake of simplicity, that the positive charge numbers are at the beginning of the list and the negative charge numbers follow the positive ones):

$$\sum_{\alpha=1}^{m} v_\alpha \left(x_{31}^{v_\alpha/v_1} - x_{42}^{v_\alpha/v_1} x_{21}^{v_\alpha/v_1} \right) C_{\alpha,1} + \sum_{\alpha=m+1}^{s} v_\alpha \left(x_{31}^{v_\alpha/v_1} - x_{42}^{v_\alpha/v_1} x_{21}^{v_\alpha/v_1} \right) C_{\alpha,1} = 0 \tag{C8}$$

and:

$$\sum_{\alpha=1}^{m} v_\alpha \left(x_{31}^{v_\alpha/v_1} - x_{42}^{v_\alpha/v_1} x_{21}^{v_\alpha/v_1} \right) C_{\alpha,1} + \sum_{\alpha=m+1}^{s} (-v_\alpha) x_{31}^{v_\alpha/v_1} x_{42}^{v_\alpha/v_1} x_{21}^{v_\alpha/v_1} \left(x_{31}^{-v_\alpha/v_1} - x_{42}^{-v_\alpha/v_1} x_{21}^{-v_\alpha/v_1} \right) C_{\alpha,1} = 0$$

(C9)

In equation (C9) all coefficients $v_\alpha C_{\alpha,1}$ for positive v_α and $(-v_\alpha) x_{31}^{v_\alpha/v_1} x_{42}^{v_\alpha/v_1} x_{21}^{v_\alpha/v_1} C_{\alpha,1}$ for negative v_α are positive, whereas the expressions $\left(x_{31}^{v_\alpha/v_1} - x_{42}^{v_\alpha/v_1} x_{21}^{v_\alpha/v_1} \right)$ for positive v_α and $\left(x_{31}^{-v_\alpha/v_1} - x_{42}^{-v_\alpha/v_1} x_{21}^{-v_\alpha/v_1} \right)$ for negative v_α have all the same sign (are all positive or all negative), because their components are monotonically increasing functions of $x_{..}$ (if $x_{31} > x_{42} x_{21}$, then $x_{31}^{a} > x_{42}^{a} x_{21}^{a}$, and if $x_{31} < x_{42} x_{21}$, then $x_{31}^{a} < x_{42}^{a} x_{21}^{a}$, for all positive a). Thus, for equation (C9) to be valid, the following equations must hold for all α:

$$x_{31}^{v_\alpha/v_1} = x_{42}^{v_\alpha/v_1} x_{21}^{v_\alpha/v_1} \quad \text{(C10)}$$

or:

$$x_{21}^{v_\alpha/v_1} = x_{31}^{v_\alpha/v_1} / x_{42}^{v_\alpha/v_1} = x_{31}^{v_\alpha/v_1} x_{24}^{v_\alpha/v_1} \quad \text{(C11)}$$

This proves equation (24).

Finally, using equation (23), the equation (C11) can be expressed as follows:

$$\frac{C_{\alpha,2}}{C_{\alpha,1}} = \frac{C_{\alpha,3}}{C_{\alpha,1}} \frac{C_{\alpha,2}}{C_{\alpha,4}} \quad \text{(C12)}$$

which yields that $C_{\alpha,3} = C_{\alpha,4}$.
